# Supplementary material for: Work-family conflict among hotel housekeepers in the Balearic Islands (Spain)
Source: PLoS One. 2023 Mar 29;18(3):e0269074. doi: 10.1371/journal.pone.0269074 (PMC10057743; doi:10.1371/journal.pone.0269074)
Supplement: Attachment — Submitted filename: Dear Editor 11 Aug 2022.docx [file pone.0269074.s001.docx]

Dear Editor,

Authors explain the domestic burden and difficulties in work-life balance (WLB) among hotel housekeepers (HHs). The study has 1,043 hotel housekeepers in the Balearic Islands, Spain [773 from Mallorca, 89 from Menorca, 137 from Eivissa, and 44 from Formentera]. One advantage of the study is its sample size because authors mention that about 13,000 possible HHs was available from the Balearic Public Health Services. It is unique dataset gives an important value to the paper. Even though the article has merits, it has some problems. Therefore, I recommend publishing with minor revision.

Sincerely

Major Issues

1. WFC variable has five categories. Therefore, instead of using just binary logistic regressions, authors must use ordered probit models.

Minor Issues

1. In the conclusion, authors wrote that HHs is a 100% feminized occupational group. However, they must mention that their sample do not include men in the data collection part (even in the introduction).
2. Proofreading is required; for example, city names must be written in English e.g., Ibiza.
