# Supplementary material for: Work-family conflict among hotel housekeepers in the Balearic Islands (Spain)
Source: PLoS One. 2023 Mar 29;18(3):e0269074. doi: 10.1371/journal.pone.0269074 (PMC10057743; doi:10.1371/journal.pone.0269074)
Supplement: Attachment — Submitted filename: response to reviewers PONE-D-22-13784.docx [file pone.0269074.s003.docx]

*Please, find following our responses to editor and reviewers comments.*

Additional Editor Comments (if provided):

Dear Author,

This is a good paper and makes a significant contribution to understanding work life balance or conflict.

i. **Please use formal language. It will be good to have a language editor review the work.**

*We really appreciate your comments. We reviewed English and made the corrections needed.*

- *Changes made in the sentence are underscored in yellow* “Characteristics of HHs’ job –inflexible work hours, high physical demands, etc.- might represent a barrier to attend personal and family demands.”.
- *In the sentence* “Part of these differences in relation to national and international surveys might be associated to the fact that these surveys include a more heterogeneous sample regarding social class, while our study focused on a single occupational group”.

*We changed it as follows (changes are underscored in yellow):* “Part of these differences in ~~relation~~ comparison to national and international surveys might be ~~associated to the fact that~~ explained by the inclusion of a more heterogeneous sample regarding social class, while our study focused on a single occupational group.”

- *In the sentence* “Our results support the relevance of structural factors and power relations to explain WLB experiences, being employees with higher educational level *and in managerial positions those with lower WFC (60).”*

*We changed it as follows (changes are underscored in yellow)*: “Our results support the relevance of structural factors and power relations to explain WLB experiences; thus, employees with higher educational level and in managerial positions ~~those with~~ report lower WFC (60).”

- *As you suggested, the sentence* “Limitations of this study include the fact that data were collected in winter (low season in the Balearic Islands) in order to be able to reach HHs for interviews.” *was corrected (changes are underscored in yellow):* “~~Limitations of this study include the fact that~~ One of the study’s limitation is that the data were collected in winter (low season in the Balearic Islands) in order to be able to reach HHs for interviews.”
- *The sentence “*Second, due to the importance of gender in approaching to work-life interface, the fact that HHs is a 100% feminized occupational group entails that comparison with men could not be undertaken” *was corrected as follows (underscored in yellow)*: *“*Second, ~~due to~~ despite the importance of gender in approaching the work-life interface, ~~the fact that~~ comparison with men could not be undertaken because HHs is a 100% feminized occupational group in the sample’s context ~~entails that comparison with men could not be undertaken~~”.
- *The sentence* “In the Balearic Islands, although most HHs work 40 hours per week, in a continuous working-day and they have little options for flexible scheduling, since they are assigned a fixed amount of rooms that must be cleaned every day.” *was corrected as follows (changes are underscored in yellow):* “In the Balearic Islands, although most HHs work 40 hours per week, in a continuous working-day and ~~they~~ have little options for flexible scheduling, ~~since~~ they are assigned a fixed amount of rooms that must be cleaned every day.”
- *The sentence:* “WLB is also framed by institutional context, such as the welfare state regime. Spain has been labelled as a Southern welfare state regime -coupled with Italy, Greece and Portugal-, characterized by low levels of social expenditure; higher proportion of population at risk of poverty; higher inequality levels; the presence of strong ‘familism’, a term to describe that family (women) has a central role in providing care work (38,40); and the underdevelopment of family benefits and services (38,41).” *was edited as follows* *(underscored in yellow)*: “WLB is also framed by institutional context, such as the welfare state regime. Spain has been labelled as a Southern welfare state regime –~~coupled~~ along with Italy, Greece and Portugal-, characterized by low levels of social expenditure; higher proportion of the population at risk of poverty; higher inequality levels; the presence of strong ‘familism’, a term to describe that family (women) has a central role in providing care work (38,40); and the underdevelopment of family benefits and services (38,41).”

*Other minor changes and corrections were made and were marked with track changes tool.*

ii. **Change most of the very old references.**

*Thank you for your comment. We checked the outdated references (all those before 2010) and we replaced for others more contemporary. Following we explain in detail the references changed:*

*Reference number 6 (in the previous version of the manuscript): Carlson DS, Grzywacz JG, Zivnuska S. Is work-family balance more than conflict enrichment? Hum Relations. 2009;62(10):1-20.*

*It was replaced by:*

14.	Mas-Machuca M, Berbegal-Mirabent J, Alegre I. Work-life balance and its relationship with organizational pride and job satisfaction. J of Manag Psychol. 2016;31(2):586-602.

15.	Jia CX, Cheung CK, Fu C. Work support, role stress, and life satisfaction among chinese social workers: The mediation role of work-family conflict. Int J Environ Res Public Health. 2020;17(23):1–14.

*Reference number 9 (in the previous version of the manuscript): Krause N,Scherzer T, Rugulies R. Physical workpload, work intensification, and prevalence of pain in low wage workers: Results from a participatory research project with hotel room cleaners in Las Vegas. AM J Ind Med. 2005 Nov;48(5):326-37.*

It was replaced by:

18.	Hsieh Y-C (Jerrie), Apostolopoulos Y, Hatzudis K, Sönmez S. Occupational exposures and health outcomes among Latina hotel cleaners. Hisp Health Care Int. 2014;12(1):6–15.

*Reference number 25 (in the previous version of the manuscript): Directorate-General for Employment Social Affairs and Inclusion (European Comission). One hundred words for equality. A glossary of terms on equality between women an men. 1998.*

*It was replaced by:*

34.	UNICEF Regional Office for South Asia. Gender equality: Glossary of terms and concepts. 2017. Available from: [https://www.unicef.org/rosa/media/1761/file/Gender glossary of terms and concepts .pdf](https://www.unicef.org/rosa/media/1761/file/Gender%20glossary%20of%20terms%20and%20concepts%20.pdf)

*Accordingly, we also changed the sentence cited as follows:* “a “disproportionate difference between men and women and boys and girls, particularly as reflected in attainment of development goals, access to resources and levels of participation”.

*Reference number 27 (in the previous version of the manuscript): Brines J. Economic dependency, gender, and the division of labor at home. Am J Sociol. 1994;100(3).*

*It was replaced by:*

36.	Perry-Jenkins M, Gerstel N. Work and family in the second decade of the 21st Century. J Marriage Fam. 2020;82(1):420–53.

*References 31 and 32 (in the previous version of the manuscript): 31. Casper WJ, Bordeaux C, ELT, Lockwood A, Lambert D. A review of research methods in IO/OB work-family research. J Appl Psychol. 2007;92(1):28-43.* AND *32. Chang A, McDonald P, Burton PM. Methodological choices in work-life balance research 1987 to 2006: a critical review. Int J Hum Resour Manag. 2009;12(November):25-7.*

*These references were removed and were not replaced because we did not find any more contemporary ones and because the content was sustained enough with the third reference in this paragraph (number 39 in the new version of the manuscript). So the paragrpah (the last one before ‘Materials and Methods’ section) in which these two references were is in the version reviewed as follows:*

“Considering that most studies on WFC have not focused on working class or unskilled workers [39], and the importance of the intersection of gender and social class, this study contributes to filling the gap on WFC and working class in the literature.”

*Reference 34 (in the previous version of the manuscript): Ferrera M. Reconstructing the welfare state in Southern Europe. Surviv Eur Welf State. 2000;166-82.*

*This reference was removed. We considered that there were another two references in this sentence and the idea was well sustained with the remaining references. The sentence in the reviewed version is as follows (underscored in yellow):*

“WLB is also framed by institutional context, such as the welfare state regime. Spain has been labelled as a Southern welfare state regime -~~coupled~~ along with Italy, Greece and Portugal-, characterized by low levels of social expenditure; higher proportion of the population at risk of poverty; higher inequality levels; the presence of strong ‘familism’, a term to describe that family (women) has a central role in providing care work [38,40]; and the underdevelopment of family benefits and services [38,41].”

*Reference number 37 (in the previous version of the manuscript): Leon M. Welfare state regimes and the social organization of labour: Childcare arrangements and the work/family balance dilemma. Sociol Rev. 2005;53(SUPPL.2): 204-18.*

*It was replaced by:*

43.	Quashie NT, Wagner M, Verbakel E, Deindl C. Socioeconomic differences in informal caregiving in Europe. Eur J Ageing. 2022;19(3):621–32.

*We also modified the sentence associated to reference* Leon M.(2005)*:*

*The original sentence was:* “In face of that, “individual informal arrengements are the most common stratey to reconcile work and family life (35,37)”.

*The sentence in the reviewed version is as follows:* “In face of that, informal caregiving provided by families (mainly, by women) is a frequent strategy to attend care needs and reconcile work and family life [41,43]”.

*Reference number 50 (in the previous version of the manuscript): González MJ, Jurado-guerrero T, Naldini M. What made him change? An individual and national analysis of men’s participation in housework in 26 countries. DemoSoc Working Paper. Barcelona; 2009. Report No.: 2009—30.*

*It was replaced by:*

1.	Bianchi SM, Sayer LC, Milkie MA, Robinson JP. Housework: Who did, does or will do it, and how much does it matter? Soc Forces. 2012;91(September):55–63.

*Reference numbre 51 (in the previous version of the manuscript): Byron K. A meta-analytic review of work-family confict and its antecedents. J Vocat Behav. 2005;67(2):169-98*

*It was replaced by:*

57.	Ford MT. Linking household income and work-family conflict: A moderated mediation study. Stress Heal. 2011;27:144–62.

58.	Rubenstein AL, Morrison HM, Whiting SW, Bosco FA. More money, more problems? An examination of the dynamic relationship between income and work-family conflict. J Occup Organ Psychol. 2022;95:305–31.

*References 52 and 53 (in the previous version of the manuscript): 52. Kossek EE, Ozeki C, Hills D. Work-family conflict, policies, and the job-life satisfaction relationship: A review and directions for organizational behavior-human resources. J Appl Psychol. 1998;83 (April):139-49.*AND *53. Netemeyer RG, Boles JS. Development and validation of work-family conflict and family-work conflict scales. J Appl Psychol. 1996;81(4):400-10.*

*They were replaced by:*

57.	Ford MT. Linking household income and work-family conflict: A moderated mediation study. Stress Heal. 2011;27:144–62.

58.	Rubenstein AL, Morrison HM, Whiting SW, Bosco FA. More money, more problems? An examination of the dynamic relationship between income and work-family conflict. J Occup Organ Psychol. 2022;95:305–31.

*We did not removed some other references dated before 2010 because they were referring to questionnaires used in the study or its validation process, or to definitions of key concepts, or classical articles in this field. These references are:*

11.	Greenhaus JH, Beutell NJ. Sources of conflict between work and family roles. Acad Manag Rev. 2011;10(1):76–88.

20.	Rotter JB. Generalized expectancies for internal versus external control of reinforcement. Psychol Monogr Gen Appl. 1966;80(1):1–28.

25.	Thoits PA. Conceptual, methodological, and theoretical problems in studying social support as a buffer against life stress. J Heal Soc Behav. 1982;23(June):145–59.

48.	Broadhead WE, Gehlbach SH, Gruy F V, Kaplan BH. The Duke-UNC functional social support questionnaire. Measurement of social support in family medicine patients. Med Care. 1988;26(7):709–23.

49.	de la Revilla Ahumada L, Bailón E, de Dios Luna J, Delgado A, Prados MA, Fleitas L. [Validation of a functional social support scale for use in the family doctor’s office]. Aten primaria. 1991 Oct;8(9):688–92.

50.	Bellón Saameño JA, Delgado Sánchez A, del Castillo JD, Lardelli Claret P. [Validity and reliability of the Duke-UNC-11 questionnaire of functional social support]. Aten primaria. 1996;18(4):153—6, 158—63.

iii. **Considering looking at the migration status of the respondents. You can use this demographic characteristics to explain the social support resources available to them.**

*Thank you very much for your suggestion about looking at the migration status of the participants. However, we performed the ordered probit models as suggested by Reviewer#1 and social support was not statistically significant (see p-value=0.109 for the variable “recP64_DUKE_UNC_categories_01” in the SPSS output below).*

*We also adapted the manuscript to PLOS ONE’s style requirements (we adapt headings to the format required, we added a short title, etc.) and we made sure that the reference list is complete and correct. We made sure that information regarding participant consent and consent by the ethics committee was provided. Finally, we changed the data availability statement as follows: “The anonymized database set is available at* [*https://doi.org/10.5281/zenodo.7509974*](https://doi.org/10.5281/zenodo.7509974)*”.*

**Reviewer #1**

**Major Issues**

1. **WFC variable has five categories. Therefore, instead of using just binary logistic regressions, authors must use ordered probit models.**

*We really appreciate this comment. We performed ordered probit models with the WFC variable (with four categories, because the fifth category [do not know/do not answer] was labeled as missing). We changed table 5:*

Table 5. Results of ordered probit models predicting work-family conflict.

|  | **Work-life conflict** | |
| --- | --- | --- |
|  | **OR (CI 95%)*** | **p-value** |
| Living alone | Ref. |  |
| Living with one or two people | 1.51 (1.08-2.13) | 0.017 |
| Living with three or more people | 1.77 (1.23-2.54) | 0.002 |
| Difficulties making ends meet | 1.43 (1.24-1.65) | <0.001 |
| Having some dependant | 1.42 (1.22-1.66) | <0.001 |
| HHs is the main responsible of domestic tasks | 1.40 (1.20-1.63) | <0.001 |
| External locus of control | 1.17 (1.02-1.35) | 0.027 |
| Level of stress at work (min 1- max 7) | 1.12 (1.07-1.16) | <0.001 |
| Hours worked/week | 1.02 (1.00-1.03) | 0.013 |
| Satisfaction with their job (min 1- max 7) | 0.94 (0.90-0.98) | 0.003 |
| Satisfaction with their wage (min 1- max 7) | 0.96 (0.92-1.00) | 0.049 |
| Age (years) | 0.99 (0.98-1.00) | 0.004 |

*We changed the content of the abstract according to the new results, as follows:*

“Risk factors for perceiving difficulties in WLB were: living with someone else (regardless of the number of co-habitants), ~~low social support,~~ having difficulties making ends meet, being the main ~~responsible~~ person in charge of ~~for~~ domestic tasks, having a dependant, having an external locus of control, presenting higher levels of stress at work, working more hours a week and being younger.”

*We changed the “Dependent variables” as follows (the changes are underscored in yellow):*

“WFC was measured through the difficulties reported in WLB in response to the question “How easy or difficult is it for you to combine work with your care responsibilities?”. The respondent gave an answer of “very easy”, “rather easy”, “rather difficult”, “very difficult”, “do not know/do not answer”. In the bivariate analysis “very easy” and “rather easy” were categorized as “easy”, and “rather difficult” and “difficult” as “difficult”. This question was taken from the European Quality of Life Survey (EQLS, 2016) [6].”

*We changed the “Statistical analysis” section as follows:*

“To assess the relationship between sociodemographic and labour variables with difficulties in WLB the chi-squared test and Student’s t-test were performed. A post-hoc z-test on the adjusted residuals with Bonferroni correction was applied to detect between which categories there were significant differences.

The generalized linear model, with an ordered dependent variable and a probit link function, was performed. We initially selected predictors with a p-value <0.20 in the bivariate analysis. Final predictive variables were selected from a backward stepwise procedure; we carried out likelihood ratio test to assess the goodness of fit of the competing statistical models.

All statistical analyses were done with SPSS for Windows version 23.0 and p-values under 0.05 were considered statistically significant.”

*Also, we changed the section ‘Risk and protective factors related to work-life balance’ in ‘Results’:*

“Table 5 shows the results of the ordered probit model for difficulties in WLB. An increased risk of WFC was associated with living with one or more people, having difficulties making ends meet, taking care of any dependant, being the main person in charge of domestic tasks, having an external locus of control, higher levels of stress, working more hours a week and being younger. Factors that reduce the risk of WFC are satisfaction with their job and with their wage.”

*Additionally, in the ‘Discussion’ section we made some changes in order to adapt it to the new results:*

“Ordered probit models allowed us to control for the effect of different variables from both spheres –family/personal and work-. Results regarding factors explaining WFC showed that family demands and its characteristics (having dependants, living with one or more people, being the main person in charge of domestic tasks), having an external LOC, and economic factors (difficulties making ends meet) along with labour aspects (higher levels of stress at work, working more hours) and being younger had a statistically significant association with an increase in WFC. On the contrary, higher levels of job and wage satisfaction had a protective effect on WFC. These relationships show how labour and domestic spheres are non-separate worlds, since the characteristics of one influence the performance of the other. Results also show that WLB is beyond individual willing and is strongly influenced by economic, labour and domestic factors along with gendered allocation of care and housework.”

*We deleted the following paragraph since social support was not statistically significant in the model:*

“Our findings support that social support (at work, from family and friends) reduces WFC among HHs (18,19). HHs with higher support at the workplace, from family and friends experienced lower WFC probably because they had help to face and manage contradicting demands from work and family spheres.”

*And we included the following one instead:*

“Our findings support that LOC has a negative relationship with WFC: there are lower levels of WFC among people with an internal LOC (56). People with an internal LOC perceive more control about what happens in their life, so they might be able to better handle stressors. Also, our results pointed out that personality traits might play a role in determining WFC, along with situational and structural factors.”

*We change the conclusion as follows*:

“This study provides a detailed understanding of individual, labour, economic and family factors explaining WFC among HHs, a non-skilled 100% feminized occupational group. Risk factors of perceiving difficulties in WLB were living with one or two more people, ~~reporting low social support,~~ having difficulties making ends meet, having some dependant, being the main person ~~responsible~~ in charge of domestic tasks, having an external LOC, ~~having some dependant~~ presenting higher levels of stress at work and being younger. Protective factors from experiencing WFC were satisfaction with their job and with their wage. Thus, our findings highlight the connection between work and personal demands and resources. Additionally, results indicate that intersection between social class and gender, at least, plays a central role in explaining WFC.”

**Minor Issues**

1. **In the conclusion, authors wrote that HHs is a 100% feminized occupational group. However, they must mention that their sample do not include men in the data collection part (even in the introduction).**

*Thank you for your observation. We included a sentence in the “Data collection” section*: “All participants were female”.

2. Proofreading is required; for example, city names must be written in English e.g., Ibiza.

*Thank you for your comment.* “Eivissa” has been changed for “Ibiza” in the “Data collection” section.

**Reviewer #2**

Theoretical introduction: Well written, with a good description of work-family conflict theory and very good interconnection between theory and the population in analysis. Interesting interpretation of the context (profession and country). In general, the introduction itself is well developed, with a good description of the study goal. Could be interesting some more theoretical development about work-family balance, not specifically as an absence of WFC, but as a concept in itself.

*We really appreciate all your comments. We made the changes you suggested, as indicated following.*

*First, we included some references in the first paragraph of the article:*

“The massive incorporation of women to the labour market in the past decades raised the interest on the articulation of paid and unpaid work. Western societies have been traditionally organized around the sexual division of labour, by which men have been the main responsible partner for paid work and women for unpaid care work [1]. This division of labour has been challenged by the growth in the number of women entering the labour market [2].”

**Reference 1**: Bianchi SM, Sayer LC, Milkie MA, Robinson JP. Housework: Who did, does or will do it, and how much does it matter? Soc Forces. 2012;91(September):55–63.

**Reference 2**: Lappegård T, Goldscheider F, Bernhardt E. Introduction to the special collection on finding work-life balance: History, determinants, and consequences of new breadwinning models in the industrialized world. Demogr Res Res. 2017;37(September).

*We also included more theoretical development about the term of work-life balance. In order to do so, we changed the first two paragraphs of the introduction as following:*

“The massive incorporation of women to the labour market in the past decades raised the interest on the articulation of paid and unpaid work. Western societies have been traditionally organized around the sexual division of labour, by which men have been the main responsible partner for paid work and women for unpaid care work [1]. This division of labour has been challenged by the growth in the number of women entering the labour market [2].

The term “work-life balance” (WLB) can be defined as the ability to successfully manage different demands coming from different life domains (i.e. allocating enough time and resources, being satisfied and involved in a balanced manner across life domains) [3]. Life domains can be understood as work-related (including just paid work) and non-work related (the rest of activities: leisure, caring, learning, etc). Since demands across life domains vary during life course, WLB acquires different meanings depending on the situation of the person [4]. The importance of the concept raised during the 1960s, when a large amount of women entered the formal labour market, while remaining in charge for domestic and reproductive tasks [5]; this situation entailed a challenge to the traditional sexual division of labour [2]. Since then, WLB has been a main political and social issue related to gender (in)equality [6]; WLB of working parents and carers has been the focus of several studies (see e.g. Rapoport & Rapoport, 1965 [7]), reports and laws [8–10] in recent decades.”

Methodology: Good description of measures evaluation and methodological concepts used. Very positive number of participants, with diversity of sociodemographic variables.

Results: Well written, with a good concise presentation of tables and a logical order. Good description of results in all variables which gives a suitable overview of the sample.

Discussion: Important interconnection with theoretical framework presented throughout the manuscript. Important theoretical implications for social class and work-family conflict, extending studies regarding higher educational level positions.

Bibliography: Well-chosen, with a good range of diversity of fonts (articles, websites, national reports). Thematic of work-family conflict pertinently sustained.

General commentary: Thank you for the opportunity of reading this manuscript. It’s an important topic with an interesting, and not so-common, population on study. Important to complement and extend work-family research, specifically with unskilled population and beyond organizational 9 to 5 models.

Considering all the mentioned above, I suggest an acceptation of the manuscript, with minor improvement (e.g., work-life balance theoretical development).
